# Genes and signaling pathways involved in memory enhancement in mutant mice

**DOI:** 10.1186/1756-6606-7-43

**Published:** 2014-06-04

**Authors:** Yong-Seok Lee

**Affiliations:** 1 Department of Life Science, College of Natural Science, Chung-Ang University, Seoul 156-756, Republic of Korea

**Keywords:** Memory, Synaptic plasticity, Long-term potentiation (LTP), Hippocampus, Treatment

## Abstract

Mutant mice have been used successfully as a tool for investigating the mechanisms of memory at multiple levels, from genes to behavior. In most cases, manipulating a gene expressed in the brain impairs cognitive functions such as memory and their underlying cellular mechanisms, including synaptic plasticity. However, a remarkable number of mutations have been shown to enhance memory in mice. Understanding how to improve a system provides valuable insights into how the system works under normal conditions, because this involves understanding what the crucial components are. Therefore, more can be learned about the basic mechanisms of memory by studying mutant mice with enhanced memory. This review will summarize the genes and signaling pathways that are altered in the mutants with enhanced memory, as well as their roles in synaptic plasticity. Finally, I will discuss how knowledge of memory-enhancing mechanisms could be used to develop treatments for cognitive disorders associated with impaired plasticity.

## Introduction

**Figure 1:**
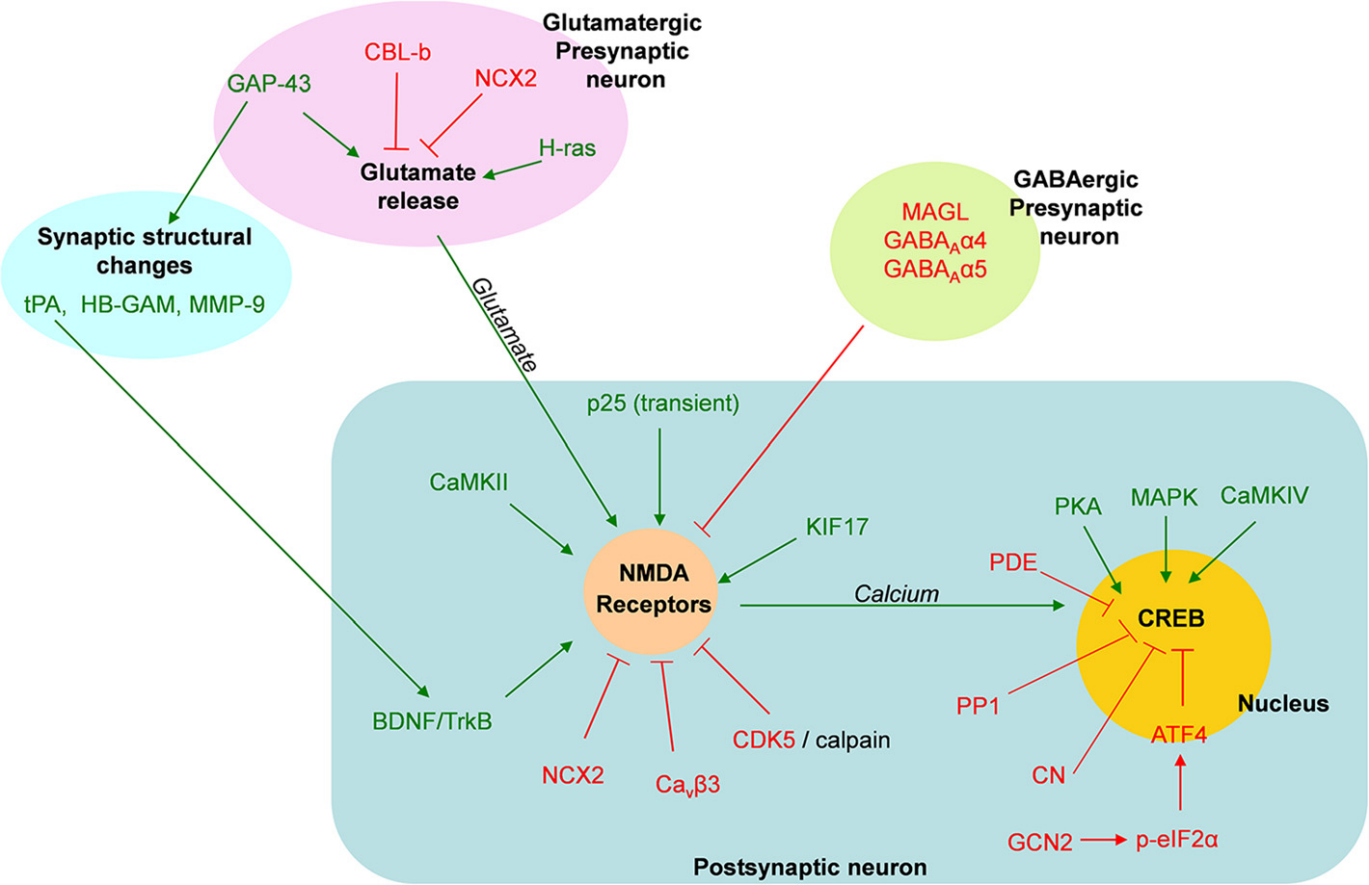
**Molecules involved in memory enhancement.** Signaling pathways in the presynaptic axonal terminal and the postsynaptic dendritic spine and nucleus are illustrated in a simplified manner. Green and red arrows indicate positive and negative regulations, respectively. Memory is enhanced either by the over expression/activation of molecules colored in green or the deletion/inhibition of molecules in red. The detailed roles of some of these molecules in LTP and memory are described in the text. Cbl-b, casitas B-lineage lymphoma-b; NCX2, Na^+^/Ca^2+^ exchanger type 2; GAP-43, growth-associated protein 43; tPA, tissue-type plasminogen activator; HB-GAM, heparin-binding growth-associated molecule; MMP-9, Matrix metallopeptidase 9; GABA, γ-aminobutyric acid; MAGL, Monoacylglycerol lipase; CaMKII, calcium calmodulin kinase II; BDNF, Brain-derived neurotrophic factor; Cdk5, Cyclin-dependent kinase 5; Cavβ3, beta intracellular subunit of the voltage-gated calcium channel; PKA, protein kinase A; PDE, phosphodiesterase; PP1, protein phosphatase 1; MAPK, mitogen-activated protein kinase; CaMKIV, calcium calmodulin kinase IV; CN, calcineurin; ATF4, activating transcription factor 4; GCN2, general control nonderepressible 2; p-eIF2α, phosphorylated eukaryotic translation initiation factor 2 subunit α.

The brain is the most complex organ in the human body, containing over 100 billion neurons, which form countless synapses. Furthermore, numerous signal transduction pathways interact with each other to build networks in every single neuron. Considering this complexity, it might seem naïve to think that manipulating a single molecule or signaling pathway in a limited area of the brain can enhance memory. Remarkably, however, it is not rare for this to happen. In the past two decades, advances in genetic engineering have permitted the generation of numerous mutant mouse lines in the field of neuroscience; these involve the transgenic overexpression, knockout (deletion), or knock-in (replacement) of specific genes. Although most of these mutant mice have impaired brain function, including learning and memory, a remarkable number show memory enhancement, as reviewed in this article (Figure 1, Table 1).

**Table 1:** Mutant mice with enhanced memory

**Mutant**	**Memory phenotypes**	**LTP phenotypes**	**References**
***Excitatory synaptic transmission***			
*NR2B (GluN2B)* Tg	Enhanced in MWM, CFC, ORT, NMT	Enhanced CA1 LTP	[1-4]
*Cdk5* cKO	Enhanced in CFC, reversal learning in MWM	Enhanced CA1 LTP	[5]
*p25* Tg	Enhanced in MWM, CFC	Enhanced CA1 LTP	[6]
*Kif17* Tg	Enhanced in MWM, DMT	Not determined	[7]
*ORL1* KO	Enhanced in MWM, CFC, PA	Enhanced CA1 LTP	[8,9]
*Hgf* Tg	Enhanced in MWM	Not determined	[10]
*Ca*_*v*_*β3* KO	Enhanced in MWM	Enhanced CA1 LTP	[11]
*Dao* KO	Enhanced in MWM	Enhanced CA1 LTP	[12]
***Presynaptic function***			
*H-ras* Tg	Enhanced in MWM, CFC	Enhanced CA1, cortical LTP	[13]
*Ncx2* KO	Enhanced in MWM, CFC, ORT	Enhanced CA1 LTP	[14]
*Cbl-b* KO	Enhanced in MWM (remote memory)	No change in CA1 LTP	[15]
*Gap43* Tg	Enhanced in MWM	Enhanced CA1 LTP	[16]
***Inhibitory synaptic transmission***			
*GABA*_*A*_*R α4* (*Gabra4*) KO	Enhanced in CFC, TFC	Not determined	[17]
*Magl* KO	Enhanced in MWM, ORT	Enhanced CA1 LTP	[18]
*Pkr* (*Eif2ak2*) KO	Enhanced in MWM, CFC, AFC	Enhanced CA1 LTP	[19]
*GABA*_*A*_*R α5* (*Gabra5*) KO	Enhanced in MWM	Trend of enhanced CA1 LTP	[20]
*Grpr* KO	Enhanced in CFC, AFC	Enhanced amygdala LTP	[21]
***Network activity***			
*Bec1* KO	Enhanced in MWM, YM	No change in CA1 LTP; Impaired LTP in Tg	[22]
*Kvβ1.1* KO	Enhanced in MWM (aged mice only)	Enhanced CA1 LTP (aged mice only)	[23]
*Hcn1* KO	Enhanced in MWM	Enhanced perforant path LTP	[24]
***Transcriptional regulation and its upstream molecules***			
CREB-Y134F Tg	Enhanced in MWM, CFC, SR, CD	Enhanced CA1 LTP	[25]
CREB-DIEDML Tg	Enhanced in CFC, SR	Not determined	[25]
eIF2α^S51A^ KI	Enhanced in MWM, CFC, AFC	Enhanced CA1 LTP	[26]
*Gcn2* KO	Enhanced in MWM, impaired in CFC	Enhanced CA1 LTP	[27]
ATF4, C/EBP CI	Enhanced in MWM	Enhanced CA1 LTP	[28]
*CamkIV* Tg	Enhanced in CFC	Enhanced CA1 LTP	[29]
*Ac1* Tg	Enhanced in ORT	Enhanced CA1 LTP	[30]
*Ap oa*_*1*_ Tg	Enhanced in CFC, ORT	Enhanced CA1 LTP	[31]
*Pde4d* KO	Enhanced in MWM, RAM, ORT	Not determined, but see [32]	[33]
*Pde8b* KO	Enhanced in MWM, CFC	Not determined	[34]
Calcineurin CI	Enhanced in MWM, AFC, ORT	Enhanced CA1 LTP	[35,36]
PP1 CI	Enhanced in MWM, ORT	Enhanced CA1 LTP	[37,38]
***Translational regulation***			
*Paip2a* KO	Enhanced in MWM, OLT, CFC	Enhanced CA1 L-LTP	[39]
*Fkbp12* KO	Enhanced in CFC	Enhanced CA1 L-LTP	[40]
***Epigenetic regulation***			
*Hdac2* KO	Enhanced in CFC, AFC, NMT	Enhanced CA1 LTP	[41]
***miRNA biogenesis***			
*Dicer1* KO	Enhanced in MWM, CFC, TFC	Enhanced CA1 LTP	[42]
***Extracellular molecules***			
*Mmp9* Tg	Enhanced in MWM, ORT	Enhanced CA1 LTP	[43]
*tPA* (*Plat*) Tg	Enhanced in MWM	Enhanced CA1 LTP	[44]
*HB-GAM* (*Ptn*) Tg	Enhanced in MWM	Enhanced CA1 LTP	[45,46]
***Other manipulations***			
*Ncs-1* Tg	Enhanced in MWM, ORT	Enhanced perforant path LTP	[47]
*Rgs14* KO	Enhanced in MWM (learning), ORT	Enhanced CA2 LTP	[48]
*5-HT*_*3*_*R* Tg	Enhanced in CFC	Not determined	[49]
*Maoa* KO	Enhanced in CFC, AFC	Not determined	[50]
*Hdc* KO	Enhanced in MWM, CFC, AFC	Enhanced CA1 LTP	[51,52]
*Def45* KO	Enhanced in MWM, ORT	Not determined	[53,54]
*EC-SOD* Tg	Enhanced in MWM, impaired CFC	Enhanced CA1 LTP	[55]
*S100b* KO	Enhanced in MWM, CFC	Enhanced CA1 LTP	[56]

Tg, transgenic; KO, knockout; KI, knock-in; cKO, conditional KO; CI, conditional inhibition; MWM, Morris water maze; CFC, contextual fear conditioning; AFC, auditory fear conditioning; TFC, trace fear conditioning; ORT, object recognition test; OLT, object location test; RAM, radial arm maze; SR, social recognition; DMT, delay matching to place task; NMT, non-match to place task; YM, Y-maze; L-LTP, late phase LTP.

Assessing higher cognitive functions such as memory in rodents became possible due to the development of diverse animal behavioral tasks. For example, several tasks examine hippocampus-dependent memory in rodents. The Morris water maze and contextual fear conditioning are the most commonly used hippocampal-dependent tasks. In the Morris water maze, mice are trained to find and remember the location of a platform that is hidden under the water, using spatial cues in the test room. Contextual fear conditioning is a form of associative learning test in which the animals associate the given context (training chamber) with noxious stimuli (foot shocks).

Mutant mice have also been used extensively to study the role of genes and signaling pathways involved in synaptic plasticity. Following the first report of long-term potentiation (LTP) in the dentate gyrus of the hippocampus by Bliss, Lømo and Gardner-Medwin in 1973 [57,58], the idea that long-term synaptic plasticity is a cellular mechanism essential to learning and memory has been supported and also challenged by a large body of literature [59-63]. However, recent studies strongly suggest that such long-lasting changes are indeed induced by learning in the hippocampus and amygdala [64-66].

In this article, the genes and signaling pathways that have been successfully manipulated to enhance memory in mutant mice will be reviewed. In parallel, the correlation between enhanced memory and increased LTP will also be discussed to argue that this form of synaptic plasticity plays a critical role in learning and memory.

### Manipulating excitatory synaptic transmission

#### Overexpression of NR2B (GluN2B)

The *N*-methyl-d-aspartate (NMDA) receptors (NMDARs) are considered to be coincidence detectors that can associate two separate events in the brain, since they require two coincident events for activation: binding of glutamate and removal of Mg^2+^ by membrane depolarization [59,63,67]. The subsequent Ca^2+^ influx activates a variety of signaling molecules including α-Ca^2+^/calmodulin-dependent kinase II (αCaMKII) [59,62,68,69]. NMDAR comprises an obligatory subunit, NR1 (GluN1), and other subunits such as NR2 (GluN2A, 2B, 2C, and 2D) and NR3 (GluN3A and 3B) [67,70,71]. In the 1980s, Morris and his colleagues demonstrated the critical role of NMDAR in learning and LTP by showing that intraventricular infusion of the NMDAR blocker AP5 impaired spatial learning and LTP [72]. Later, this classical pharmacological experiment was revisited using elegant gene-knockout technology. Hippocampal CA1-specific deletion of NR1 impaired LTP and spatial memory without causing any confounding non-spatial learning ability deficit [73]. In addition, deletion of NR1 in the CA3 and dentate gyrus hippocampal subregions also impaired memory, though affecting its different aspects [74-76]. Among NR2 (GluN2) subunits, NR2A (GluN2A) is predominantly expressed and its deletion causes deficits in hippocampal LTP and hippocampus-dependent learning tasks [77,78]. The expression of another subunit, NR2B (GluN2B), is decreased during development and the duration of NR2B (GluN2B)-mediated currents is longer than that of NR2A (GluN2A)-mediated currents, which can allow more Ca^2+^ influx through NMDARs [71].

Tsien, Zhuo, Liu, and their colleagues generated a transgenic mouse overexpressing NR2B in the forebrain to study its role in memory and synaptic plasticity [1]. Prolonged NMDAR activation led to enhancement of LTP in the hippocampus of the NR2B transgenic mouse, and these mutants outperformed their wild type littermates in several learning and memory tasks. Firstly, the transgenic mice showed better performance than wild type littermates in the hidden-platform version of the water maze [1,79]. Secondly, both contextual and cued fear memory were enhanced in the NR2B transgenic mice. In addition, fear extinction, which is also NMDAR-dependent and thought to be another form of active learning, was also facilitated in the mutants. It was later shown that this superiority in learning and memory is retained in aged transgenic mice [2]. The memory enhancing effects of NR2B overexpression go beyond hippocampus-dependent tasks. NR2B transgenic mice also showed enhanced working memory performance in the delay non-match-to-place T-maze task [3]. Accordingly, cortical LTP was also found to be enhanced in NR2B mutant mice [3]. Recently, Wang and colleagues overexpressed NR2B in the forebrains of rats and found that these rats showed enhanced memory and hippocampal LTP, which demonstrates that the up-regulation of NR2B results in memory enhancement in multiple species [4].

#### Post-translational regulation of NR2B

The function of proteins can be regulated by multiple post-translational processes such as covalent modifications, subcellular localization, and degradation. NR2B is transported along microtubules by a neuron-specific motor protein kinesin family protein 17 (KIF17) [80]. Interestingly, overexpression of KIF17 increased the synaptic expression of NR2B and enhanced spatial and working memory in transgenic mice [7], suggesting that enhanced transport of NR2B could be the cause of the enhanced learning ability, at least to some degree.

Calpain is activated by Ca^2+^ entering through NMDARs, and it rapidly cleaves NMDAR subunits, which are clustered with a scaffolding-protein complex that contains PSD-95; subsequently, it decreases functional NMDAR expression [81,82]. The proteolysis of NR2B by calpain is accelerated by interaction of the latter with Cdk5 [5]. Hawasli and colleagues generated a conditional knockout of Cdk5 in mice and found that the deletion of Cdk5 in the adult forebrain enhances contextual fear conditioning, fear extinction, reversal learning in the water maze, and LTP [5]. In addition, Cdk5 deletion was associated with a reduction of NR2B degradation that resulted in augmentation of NMDAR-mediated currents. Recently, disrupting the NR2B-Cdk5 interaction via a small interfering peptide has been shown to increase NR2B surface levels, facilitate synaptic transmission, and subsequently, improve memory in contextual fear conditioning [83]. Consistent with this, chronic activation of p25, a strong activator of Cdk5, resulted in deficits in learning and synaptic plasticity [6,84]. However, paradoxically, the transient expression of p25 in mouse forebrain enhanced synaptic plasticity and hippocampus-dependent memory, including contextual fear conditioning and learning in the Morris water maze [6]. This memory enhancement was accompanied by increases in numbers of dendritic spines and synapses [6]. While both NR2A phosphorylation and NMDA-mediated currents are shown to increase after the transient overexpression of p25 [6], neither NR2B expression nor NR2B-mediated currents have been examined in this conditional transgenic mutant.

#### Other mutants with enhanced NMDAR function

Other manipulations have indirectly enhanced NMDAR function and subsequently enhanced memory. Mice lacking the nociception receptor, opioid receptor-like-1 (ORL1), showed enhanced learning and memory in the Morris water maze, passive avoidance task, and contextual fear conditioning [8,9]. Moreover, LTP was significantly enhanced in this mutant. Since ORL1 was initially reported to inhibit adenylyl cyclase via G proteins [85], an increased level of cAMP was postulated as the underlying mechanism for enhanced plasticity and learning in ORL1 knockout mice. However, a recent study showed that the deletion of ORL1 increases CaMKII activity and enhances NMDAR function, suggesting that enhanced NMDAR function might be responsible for the enhanced cognition in ORL1 knockout mice [8].

Transgenic mice overexpressing hepatocyte growth factor (HGF) also showed enhanced learning and memory performance in the Morris water maze [10]. Biochemical analysis revealed that NR2A and NR2B expression was significantly increased in the hippocampus of HGF transgenic mice. Taken together, these studies demonstrate that up-regulation of NMDAR function is one of the common molecular mechanisms for genetic enhancement of learning and memory. Moreover, these studies support the idea that NMDAR-dependent LTP is strongly associated with memory performance in a diverse set of behavioral tasks.

#### Enhancing excitatory presynaptic function

Studies of mice expressing a constitutively active form of H-ras (H-ras^G12V^) in axons of pyramidal neurons in the post-natal hippocampus revealed a role for Ras signaling in presynaptic neurotransmitter release in LTP and memory [13]. Presynaptic expression of H-ras^G12V^ resulted in increased activation of mitogen-activated protein kinase (MAPK) and phosphorylation of its presynaptic substrate, synapsin I. In addition, these mutants showed a number of other convergent presynaptic phenotypes, including a higher number of docked vesicles, an increased frequency of mEPSCs, and altered paired-pulse facilitation. Moreover, both hippocampal LTP and hippocampal memory were enhanced in the transgenic mice [13]. Importantly, a synapsin I mutation, which alone had no measurable effect on LTP or learning, reversed the physiological and behavioral enhancements of the H-ras^G12V^ mice, indicating that H-Ras/MAPK-dependent phosphorylation of synapsin I played a key role in the learning enhancements of these mutants. These results provide strong evidence that the learning enhancements described were caused by increased phosphorylation of synapsin I, and the subsequent enhancement of excitatory presynaptic function. Recently, Kaneko and colleagues showed that the rate of change in ocular dominance in response to monocular deprivation is accelerated in the mutants, as well as the rate of the recovery from the deprivation [86]. In addition, presynaptic LTP is enhanced in the primary visual cortex of developing H-ras^G12V^ mice (P26-30) [86].

### Manipulating inhibition

Disruption of the excitation/inhibition balance has been proposed as a mechanism underlying many psychiatric disorders such as Schizophrenia, autism, and learning disabilities [87-89]. However, evidence is accumulating that memory can be enhanced by modulating inhibition. For example, pharmacological suppression of inhibition has been shown to enhance memory consolidation [90,91]. Moreover, a picrotoxin treatment that reduces inhibitory synaptic transmission lowered the threshold for LTP induction [92]. The effect of genetic reduction of tonic inhibition was examined in GABA_A_R α4 subunit knockout mice [17]. GABA_A_R α4 knockouts showed enhanced trace and contextual fear conditioning, suggesting that reducing tonic inhibition can enhance hippocampus-dependent forms of memory [17]. LTP and other forms of synaptic plasticity have yet to be tested in this mutant.

Beyond direct manipulation of the GABA receptor, some manipulations enhance memory by indirectly affecting inhibition. Monoacylglycerol lipase (MAGL) is one of the enzymes that regulate endocannabinoid (eCB) signaling by hydrolyzing the eCB 2-arachidonoylglycerol (2-AG). Deletion of MAGL increases the level of 2-AG in the brains of MAGL knockout mice [18]. Exogenous cannabinoids impair learning and memory. In contrast, increasing the levels of 2-AG in the MAGL knockout mice enhanced learning and memory in the Morris water maze and object recognition tasks [18]. This cognitive enhancement is also accompanied by enhanced LTP in the hippocampus [18]. The cannabinoid receptor CB1 is predominantly expressed in inhibitory neurons and therefore, eCBs are implicated in the regulation of inhibitory synaptic transmission. Electrophysiological recordings suggest that the LTP enhancement in MAGL-null mutants is mediated by 2-AG-induced suppression of inhibition [18].

GABA release can be inhibited by interferon-γ, whose translation is negatively regulated by a double-stranded RNA-activated protein kinase (PKR) and eIF2α. In PKR knockout mice, the increased translation of interferon-γ has been shown to increase neuronal activity in the hippocampus by reducing GABA release [19,93]. Accordingly, GABAergic inhibition was decreased in PKR knockout mice [19]. PKR knockouts showed memory enhancement in the Morris water maze, and contextual and auditory fear conditioning. Memory extinction in contextual fear conditioning was also enhanced in PKR mutants [19]. Network excitability was increased in hippocampal slices from PKR knockout mice due to reduced GABAergic inhibition [19].

One of the key molecules for regulating neuronal excitability is the potassium channel. BEC1 (KCNH3) is a member of the ether-a-go-go (KCNH) family of voltage-gated K^+^ channels that is preferentially expressed in the forebrain. Deletion of BEC1 increased the excitability of hippocampal pyramidal neurons and enhanced working memory, spatial memory, and attention [22]. In contrast, overexpression of BEC1 in the forebrain impaired memory and LTP [22]. Taken together, these results suggest that manipulating the balance of excitation/inhibition by either reducing inhibition or increasing network excitability within an appropriate range might be a promising strategy for cognitive enhancement.

### Manipulating transcriptional regulation

#### Enhancing CREB function

The formation of long-term memories requires synthesis of new mRNA and proteins. Synaptic stimulation or learning activates multiple signaling pathways to orchestrate gene transcription and translation. Accordingly, overexpression of transcriptional activators such as CCAAT enhancer-binding protein (C/EBP) and LAPS18-like protein (LLP) has been shown to lower the threshold for long-term synaptic plasticity in the marine snail *Aplysia*[94,95].

cAMP response element-binding protein (CREB), a basic leucine zipper transcription factor, is critically involved in long-term plasticity and memory in both invertebrates and vertebrates [60,96-102]. In mammals, CREB-deficient mice have impaired LTP and long-term memory [96]. By contrast, the threshold for late phase LTP (L-LTP) was lowered in the hippocampus of mice expressing the constitutively active form of CREB (VP16-CREB) [103]. Kida and colleagues generated two transgenic mouse lines expressing dominant active CREB mutations in the forebrain: CREB-Y134F, which displays a higher affinity for PKA and CREB-DIEDML, which constitutively interacts with CBP [25]. CREB-Y134F mutant mice outperformed their wild type littermates in social recognition, contextual fear conditioning, context discrimination, and the Morris water maze task. Similarly, the CREB-DIEDML mutant also showed enhanced memory in social recognition and contextual fear conditioning, which are hippocampus-dependent tasks [25]. LTP was also enhanced in CREB-Y134F mutant mice, demonstrating that enhancing CREB function facilitates both synaptic plasticity and memory [25].

CREB activity can be modulated by other regulatory proteins [104]. Overexpression of CREB2, which is a negative regulator of CREB in *Aplysia*, blocked long-term facilitation, while inhibiting CREB2 lowered the threshold for long-term facilitation induced by serotonin treatments [105,106]. This suggests that the balance between positive and negative regulation of CREB can determine the polarity and/or strength of synaptic plasticity and memory both in invertebrates and vertebrates. Activating transcription factor-4 (ATF4), a mammalian homolog of *Aplysia* CREB2, is a negative regulator of CREB in vertebrates [107]. Chen and colleagues found that the forebrain-specific expression of a broad-spectrum dominant negative inhibitor of the C/EBP family (EGFP-AZIP) suppresses ATF4 expression [28]. This manipulation shifted the transcriptional balance in favor of activation of CREB-downstream genes and lowered the threshold for LTP and memory formation [28]. Mutant mice showed enhanced learning when they were trained in the Morris water maze using a relatively weak training protocol, and a single train of tetanus, which normally induces only E-LTP, could induce transcription-dependent L-LTP in the mutants [28]. These data suggest that relief of transcriptional repression can be an evolutionarily conserved strategy for enhancing learning and memory.

Phosphorylation of the α-subunit of eIF2 can stimulate the translation of ATF4 mRNA [108,109]. Deletion of GCN2, a conserved eIF2α kinase, has been shown to reduce the phosphorylation of eIF2α and suppress the translation of ATF4 mRNA [27]. The threshold for L-LTP was lowered and spatial memory was enhanced by this manipulation when the mutants were trained using a weak training protocol [27].

To directly examine the role of eIF2α phosphorylation in synaptic plasticity and memory, eIF2α heterozygous knock-in mice (eIF2α^+/S51A^) were generated, in which the phosphorylation of eIF2α is blocked [26]. In this mutant, the protein level of ATF4 was significantly reduced. Similarly to GCN2 knockout mice, the threshold for L-LTP was lowered and the mutants showed improved learning and memory in a variety of behavioral tasks, including contextual and cued fear conditioning, conditioned taste aversion, and latent inhibition [26].

#### Positive regulators of CREB

Adenylyl cyclases (ACs) play crucial roles in synaptic plasticity and memory in the mammalian brain by coupling Ca^2+^ currents through NMDARs to cAMP signaling [110]. Of the ACs, AC1 and AC8 are neuron-specific [110]. Wang and colleagues overexpressed AC1 in mouse forebrain to examine whether up-regulation of AC1 can enhance memory [30]. AC1 overexpression enhanced LTP, and AC1 transgenic mice showed enhanced memory in the object recognition task. After training in the object recognition task, activation of MAPK and CREB was significantly higher in mutants compared to the control mice, supporting the hypothesis that up-regulation of CREB function is associated with memory enhancement [30].

Isiegas and colleagues developed a novel transgenic system that can conditionally activate cAMP signaling in mouse forebrain by overexpressing a G_s_-coupled octopamine receptor cloned from *Aplysia*[31,111]. Administration of octopamine, which is not endogenously expressed in the mammalian nervous system, activated cAMP signaling and enhanced hippocampal LTP and memory in the object recognition task [31].

#### Negative regulators of CREB

Phosphodiesterases (PDEs) hydrolyze cAMP, and there are 11 families of PDEs in mammals. Among the four subtypes of PDE4s (A, B, C, and D), PDE4D knockout mice have shown memory enhancement in the radial arm maze, Morris water maze, and object recognition test [33]. Activation of CREB was also increased in the PDE4D knockout. Recently, deletion of PDE8B was shown to enhance memory in contextual fear conditioning, the Morris water maze, and an appetitive instrumental conditioning task [34].

### Manipulating translational regulation

Translation of protein can be regulated at multiple levels, from the initiation of translation to protein degradation. PABP-interacting protein 2 (PAIP2) negatively regulates translational initiation by inhibiting poly (A)-binding protein (PABP). PAIP2A was found to be degraded in response to either neural activation or behavioral training in contextual fear conditioning, suggesting that PAIP2A might be a negative regulator of plasticity and learning [39]. To examine whether PAIP2A is indeed involved in memory formation, Khoutorsky and colleagues generated PAIP2A knockout mice [39]. PAIP2A knockouts showed memory enhancements in multiple behavioral tasks including the Morris water maze, contextual fear conditioning, and object-location memory tasks, which all depend on the hippocampus. PAIP2A knockout mice also showed a lowered threshold for the induction of L-LTP [39].

Mammalian target of rapamycin (mTOR) signaling regulates translational initiation and is involved in memory formation and synaptic plasticity [112,113]. Dysregulation of mTOR signaling is also associated with cognitive disorders such as tuberous sclerosis [114,115]. Several mTOR-interacting proteins can regulate this signaling pathway. Among these, FK506-binding protein 12 (FKBP12) inhibits mTOR. Accordingly, activation of mTOR signaling is enhanced in brain-specific FKBP12 knockout mice and L-LTP and contextual fear memory was enhanced in the mutants [40].

### Manipulating epigenetic regulations

In addition to transcriptional and translational regulations, histone modification and DNA methylation are involved in regulating learning and synaptic plasticity. Pharmacological manipulations of histone acetylation by treating with histone deacetylase (HDAC) inhibitors have been shown to enhance memory and synaptic plasticity in both mammals and invertebrates [116,117]. In addition, reducing histone acetyltransferase activity impaired both long-term memory and LTP in mice [118,119]. Guan and colleagues generated both transgenic and knockout mice for different HDACs [41]. HDAC2-, but not HDAC1-overexpressing mice show deficits in memory and LTP, suggesting that HDAC2 might be a major target of HDAC inhibitors associated with memory enhancement [41]. Consistently, HDAC2 knockout mice showed enhanced memory and increased LTP [41]. Chromatin immunoprecipitation experiments showed that HDAC2 is associated with genes previously known to be involved in synaptic functions such as *Bdnf, Egr1, Fos, Camk2a, Creb1, Crebbp, NRXN3,* and the NMDAR subunit genes [41].

### Manipulating microRNA biogenesis

MicroRNAs (miRNAs) are small non-coding RNA molecules that inhibit the translation of their target mRNAs. The miRNAs regulate various cellular functions including synaptic plasticity, learning, and memory [120]. Dramatic changes in the expression of miRNA have been shown to occur in response to NMDA-dependent neuronal activation or behavioral training in contextual fear conditioning [121]. Dicer, a type III ribonuclease, is a key enzyme for generating the mature form of miRNAs. Konopka and colleagues investigated the effects of miRNA using conditional knockout mice expressing Cre recombinase, which is under the control of tamoxifen [42]. Deletion of Dicer1 in the adult brain reduced the abundance of mature miRNAs, but did not cause neuronal death or abnormalities in motor function, anxiety, or circadian rhythm, for up to 14 weeks after induction of the Dicer1 mutation. Interestingly, however, Dicer1 conditional knockout mice showed enhanced learning and memory in the Morris water maze and fear conditioning tests [42]. Dicer1 deletion also altered the morphology of dendritic spines and increased the levels of synaptic proteins related to plasticity, such as brain-derived neurotrophic factor (BDNF), AMPA receptor, post-synaptic density protein 95 (PSD-95), and matrix metalloproteinase-9 (MMP-9). These results suggest that miRNA might be another important player in memory enhancement [42].

### Manipulating extracellular molecules

Long-term synaptic plasticity is accompanied by synaptic structural remodeling and, therefore, manipulating any molecules that modify synaptic structures may affect synaptic plasticity and memory. MMPs are zinc-dependent proteases that are involved in the remodeling of the pericellular environment by degrading the extracellular matrix. Overexpression of MMP-9 in the forebrain enhanced memory in the Morris water maze and the object recognition task, and LTP was also enhanced in the MMP-9 transgenic mice, demonstrating that MMP-9 is a positive regulator of LTP and memory [43].

Tissue-type plasminogen activator (tPA) is an extracellular serine protease [122]. Deletion of tPA caused deficits in L-LTP and several forms of memory [123-125]. Consistently, neuronal overexpression of tPA enhanced both LTP and hippocampus-dependent spatial memory [44]. However, the mechanism underlying enhanced memory and LTP remained unclear. It has also been reported that tPA is involved in the processing of a neurotrophic factor, brain-derived neurotrophic factor (BDNF) [126]. The overexpression of tPA may increase the production of mature BDNF, which is one of the key molecules involved in L-LTP and long-term memory [127,128], with consequent enhancement of hippocampal LTP and spatial memory.

### The Role of LTP in memory enhancement

Interestingly, there are mutant mice that show enhanced LTP without changes in behavior, or even with deficits in learning and memory (Table 2). For example, PSD-95 knockout mice showed severe deficits in spatial learning and memory, whereas they showed a dramatic increase in hippocampal LTP [129]. It has been extensively debated whether LTP is both necessary and sufficient for memory storage [130]. As well as the PSD-95 knockout, the other studies summarized in Table 2 also seem to support the idea that LTP is not strongly associated with memory. This may mean that long-term synaptic plasticity such as LTP is not sufficient for long-term memory storage. So far, it is difficult to draw a firm conclusion based on the studies using mutant mice. As discussed by Neves, Cooke, and Bliss [130], in order to establish the causality of LTP for memory, one needs to design an experimental system (a mutant mouse) in which only LTP is manipulated without affecting any other biological processes. However, most of the mutant mice listed in Table 2 show other behavioral and physiological phenotypes in addition to LTP enhancement. Firstly, other synaptic structures and functions in addition to LTP are affected in the mutants. For example, deleting PSD-95 affected AMPA receptor functions [131] and dystrophin knockout altered GABAergic inhibition [132]. Secondly, genetic manipulations caused abnormalities in other physiological processes. For example, FMR2 knockout mice show increased latency to paw withdrawal in the hot-plate test, suggesting that pain perception has been altered in the mutant, which might affect the learning and memory phenotype in fear conditioning [133].

**Table 2:** LTP enhancement without memory enhancement

**Mutant**	**LTP phenotypes**	**Memory phenotypes**	**Reference**
*Psd-95* KO	Enhanced CA1 LTP	Impaired in MWM	[129]
*Limk-1* KO	Enhanced CA1 LTP	Normal initial learning, impaired reversal learning in MWM	[134]
*Syndecan-3* KO	Enhanced CA1 LTP	Impaired in MWM	[135]
*PTPδ* KO	Enhanced CA1 and CA3 LTP	Impaired in MWM, RAM	[136]
*IRSp53* KO	Enhanced CA1 LTP	Impaired in MWM, ORT	[137]
*G*_*iα1*_ KO	Enhanced CA1 LTP	Impaired in CFC, PA, ORT; but normal in MWM	[138]
*Tropomodulin-2* KO	Enhanced CA1 LTP	Impaired in CFC, MWM	[139]
*Dystrophin* KO	Enhanced CA1 LTP	Impaired in MWM, ORT	[140]
*Tsc2* KO	Enhanced CA1 LTP	Impaired in MWM, CFC^*^	[114]
*GluR2* (GluA2) KO	Enhanced CA1 LTP	Impaired in MWM	[141,142]
*Fmr2* KO	Enhanced CA1 LTP	Impaired in CFC, but normal in MWM	[133]
*dnPAK* Tg	Enhanced cortical LTP	Impaired in MWM (21 days), CFC (1 day)	[143]
*Inositol 1,4,5-triphosphate 3-kinase* KI	Enhanced CA1 LTP	No change in MWM	[144]

KO, knockout; Tg, transgenic; KI, knock-in; MWM, Morris water maze; RAM, radial arm maze; ORT, object recognition test; CFC, contextual fear conditioning; PA, passive avoidance; *impaired in contextual discrimination test.

In this review, the analysis began from the perspective of enhanced learning, not from that of enhanced LTP. As discussed above, even if LTP is enhanced by a mutation, the same genetic manipulation could cause abnormalities in other cellular processes required for normal learning and memory, which makes it more difficult to draw conclusions about the role of LTP in memory. However, a better correlation between LTP and memory was observed when LTP was analyzed in the mutants that do show memory enhancement, supporting the hypothesis that the genes and signaling pathways involved in LTP are crucial to the cellular mechanisms of memory. Moreover, bi-directional manipulations of several genes have resulted in corresponding bi-directional changes in both LTP and memory. For example, deletion of tPA in mice resulted in deficits in LTP and several forms of memory such as contextual fear conditioning, object exploration, and active avoidance tasks [123,124]. In contrast, transgenic expression of tPA enhanced both LTP and hippocampus-dependent spatial memory [44]. Bi-directional manipulations of memory phenotypes are highly correlated with similar changes in LTP in several mouse mutants that each carrying a different genetic mutation, suggesting that this form of synaptic plasticity does play a critical role in learning and memory. This review may support the concept that LTP enhancement is necessary for memory facilitation. Of the 47 mutants listed in Table 1, LTP has been examined in 38, and 36 of those 38 show enhanced LTP. Two mutants, the Cbl-b knockout and BEC1 knockout, demonstrated enhanced memory without an enhancement of LTP [15,22], which may disprove the necessity of LTP enhancement for memory enhancement. However, there are plausible explanations for this. It is well known that there are multiple protocols for LTP induction and a particular gene might only be involved in a form of LTP that is induced by a specific protocol. For example, deleting GluR-A (GluA1) caused a large deficit in the LTP induced by high frequency stimulation (100 Hz tetanus), but spared the LTP induced by a theta-burst pairing protocol [145,146]. Although the hippocampal LTP induced by high frequency stimuli was not enhanced in Cbl-b knockout mice, other protocols for enhancing LTP have not yet been tested in these mice [15]. Another possibility is that LTP enhancement might be observed in other brain areas of those mutants, because different brain areas are involved in different forms of memory. Interestingly, the spatial memory enhancement in Cbl-b knockout mice was more pronounced when memory was tested 45 days after training [15]. It has been shown that episodic memory is initially processed in the hippocampus and is then gradually transferred to the cortical areas for long-term storage [147]. Thus, long-term synaptic plasticity could be enhanced in cortical areas in Cbl-b knockout mice.

Neither high frequency nor theta-burst stimulation resulted in LTP enhancement in the hippocampus of BEC1 knockout mice [22]. Interestingly, prominent enhancements were observed in working memory and attention tests [22], which involve cellular mechanisms in other brain regions. Synaptic plasticity in other brain areas such as the prefrontal cortex has not yet been examined in this mutant. Therefore, the studies involving Cbl-b and BEC1 knockouts cannot necessarily disprove the necessity of LTP enhancement for memory enhancement. However, it is possible that the performance in memory tasks might be enhanced by LTP-independent mechanisms in some mutants. For example, increased attention or other physiological changes could be responsible for the enhancement of behavioral performance by BEC1 knockout mice in spatial and working memory tests [22].

It is worth noting that causality between a genetic manipulation and LTP enhancement is not always clear. The mechanism for each mutant summarized in this review might not be the root cause of the observed enhancements and should be regarded as tentative. For example, although the mechanism underlying memory enhancement by acute expression of p25 is categorized as altered ‘excitatory synaptic transmission’ (Table 1), this might not be the proximal cause for the memory enhancement. Instead, the impact of the mutation on synaptic structures, such as the increased number of spines, could be the direct cause for the enhancement [6]. It will also be necessary to investigate which stages of memory processing are facilitated by the smart mutations, and these are not clear in most cases.

### Potential therapeutic strategies involving memory enhancement

Elucidating memory-enhancing mechanisms will provide valuable insights into treatment options for disorders associated with memory impairments. As discussed above, reducing HDAC activity enhances memory in normal mice [41,116]. Manipulating HDAC activity has been suggested as a potential treatment for a specific cognitive disorder. Rubinstein-Taybi syndrome (RTS) is a genetic disorder associated with intellectual disabilities and skeletal abnormalities. RTS is caused by mutations in CREB binding protein (CBP), which has histone acetyltransferase (HAT) activity. Either the heterozygous deletion of *Cbp* or the expression of a mutant CBP lacking HAT activity caused deficits in LTP and memory in mice [119,148]. In these mouse models of RTS, administration of the HDAC inhibitors, SAHA or TSA, significantly improved both the behavioral and physiological phenotypes [119,148]. There is evidence that reducing the phosphorylation level of eIF2α can enhance memory in mice [26]. Interestingly, deletions of the eIF2α kinases GCN2 or PERK have been shown to prevent deficits in synaptic plasticity and spatial memory in mice expressing familial Alzheimer's disease-related mutations in APP and PSEN1 [149]. Taken together, these data suggest that genes implicated in memory enhancement in mutant mice may be potential targets for drugs designed to improve physiological conditions and behavioral outcomes in diseased brains.

### Adverse effects of mutations that enhance memory

It is important to note that unexpected and adverse behavioral effects can accompany genetic manipulations that enhance memory. For example, NR2B transgenic mice have shown enhanced chronic pain [150,151]. In addition to the hippocampus, NR2B was also overexpressed in pain-related forebrain areas of the transgenic mice, including the anterior cingulate cortex [150]. While NR2B transgenic mice and their wild-type littermates showed similar responses in an acute pain test, the NR2B transgenic mice displayed enhanced responsiveness to a peripheral injection of inflammatory stimuli that induce chronic pain, suggesting that the overexpression of NR2B in the forebrain can affect pain perception in mice [150].

Another deficit associated with memory enhancement is the loss of memory flexibility. As discussed, overexpression of AC1 enhances memory in an object recognition test [30]. However, the AC1 transgenic animals showed slower extinction for contextual fear memory [30], suggesting that this mutant might have lost its behavioral flexibility. Memory extinction does not simply involve forgetting, but is an active learning process that is critical for behavioral adaptation [152]. Similarly, while calcineurin inhibition facilitated memory in fear conditioning and conditioned taste aversion tests, it impaired the extinction of previously formed contextual fear memory and conditioned taste aversion memory [35,153]. FKBP12 knockout mice that have shown enhanced memory in contextual fear conditioning have also displayed abnormalities in other behavioral tests [40]. The mutant mice showed a performance comparable to their wild-type littermates during initial learning in the water maze and Y-maze tasks. [40]. However, the knockout mice showed deficits in reversal learning, in which the mice need to learn new locations for the escape platforms [40]. Moreover, FKBP12 mutants showed a preference for the familiar object over the novel object in an object recognition test, and displayed increased repetitive behavior in a marble-burying test [40]. These findings suggest that the other smart mutants might also display unknown behavioral or physiological side-effects.

This issue will be critical when attempts are made to develop treatments for brain disorders based on memory enhancing mechanisms. It is recommended that the researchers in this field should standardize a set of rigorous behavioral tests to examine whether a memory mutant has any other behavioral and physiological phenotypes, including changes in motor function, locomotion, pain, anxiety, social behavior, and behavioral flexibilities. Unfortunately, it is not clear why some mutations have an adverse impact on behavior. It is well known that different brain areas are involved in different aspects of learning and memory. For example, different areas are involved in consolidation and extinction of fear memory [154]. Most of the genetic manipulations described in this review affect the whole body or at least the whole forebrain of the mouse. Thus, it is not surprising that a manipulation that is beneficial to one brain function could have negative effects on another. Applying more advanced genetic techniques that are region- and/or cell-specific may help in the design of smart mice with reduced risk of side-effects.

## Conclusions

Genetic mutations that enhance memory in mice frequently result in a concomitant increase in LTP, suggesting that this form of synaptic plasticity plays a crucial role in learning and memory. Although there are still many issues to be addressed, it is clear that studies on the molecular and cellular mechanisms leading to enhanced memory in mutant mice have generated important insights into the pathways and mechanisms involved in plasticity and memory, which may help in the future development of broadly applicable approaches to treating neurological disorders.

## Abbreviations

ATF4: Activating transcription factor 4; αCaMKII: α-Ca^2+^/calmodulin-dependent kinase II; BDNF: Brain-derived neurotrophic factor; CBP: CREB binding protein; Cbl-b: Casitas B-lineage lymphoma-b; Cdk5: Cyclin-dependent kinase 5; CREB: cAMP response element-binding protein; GABA: Gamma-aminobutyric acid; GCN2: General control nonderepressible 2; eIF2α: Eukaryotic translation initiation factor 2 subunit α; HDAC: Histone deacetylase; LTP: Long-term potentiation; MAGL: Monoacylglycerol lipase; MAPK: Mitogen-activated protein kinase; miRNA: microRNA; MMPs: Matrix metalloproteinases; NMDAR: *N*-methyl-d-aspartate receptor; ORL1: Opioid receptor-like-1; PABP: Poly (A)-binding protein; PKA: Protein kinase A; PKR: RNA-activated protein kinase; tPA: Tissue-type plasminogen activator.

## Competing interests

The author declares no competing interests.
